# Validation of the Edinburgh Claudication Questionnaire in 1^st ^generation Black African-Caribbean and South Asian UK migrants: *A sub-study to the Ethnic-Echocardiographic Heart of England Screening (E-ECHOES) study*

**DOI:** 10.1186/1471-2288-11-85

**Published:** 2011-06-03

**Authors:** Philip C Bennett, Gregory YH Lip, Stanley Silverman, Andrew D Blann, Paramjit S Gill

**Affiliations:** 1 University of Birmingham Centre for Cardiovascular Sciences, City Hospital, Birmingham, B18 7QH, UK; 2 Department of Vascular Surgery, City Hospital, Birmingham, B18 7QH, UK; 3 Primary Care Clinical Sciences, University of Birmingham, Edgbaston, Birmingham B15 2TT, UK

## Abstract

**Background:**

We determined the diagnostic accuracy of the Edinburgh Claudication Questionnaire (ECQ) in 1^st ^generation Black African-Caribbean UK migrants as previous diagnostic questionnaires have been found to be less accurate in this population. We also determined the diagnostic accuracy of translated versions of the ECQ in 1^st ^generation South Asian UK migrants, as this has not been investigated before.

**Methods:**

Subjects were recruited from the Ethnic-Echocardiographic Heart of England Screening (E-ECHOES) study, a community based screening survey for heart failure in minority ethnic groups. Translated versions of the ECQ were prepared following a recognised protocol. All participants attending screening between October 2007 and February 2009 were asked to complete the ECQ in the language of their choice (English, Punjabi, Bengali, Urdu, Hindi or Gujarati). Subjects answering positively to experiencing leg pain or discomfort on walking were asked to return to have Ankle Brachial Pressure Index (ABPI) measured.

**Results:**

154 out of 2831 subjects participating in E-ECHOES (5.4%) were eligible to participate in this sub-study, for which 74.3% returned for ABPI assessment. Non-responders were younger than participants (59[9] vs. 65[11] years; p = 0.015). Punjabi, English and Bengali questionnaires identified participants with Intermittent Claudication, so these questionnaires were assessed. The sensitivities (SN), specificities (SP), positive (PPV) and negative (NPV) predictive values were calculated. English: SN: 50%; SP: 68%; PPV: 43%; NPV: 74%. Punjabi: SN: 50%; SP: 87%; PPV: 43%; NPV: 90%. Bengali: SN: 33%; SP: 50%; PPV: 13%; NPV: 73%. There were significant differences in diagnostic accuracy between the 3 versions (Punjabi: 83.8%; Bengali: 45%; English: 62.2%; p < 0.0001). No significant differences were found in sensitivity and specificity between illiterate and literate participants in any of the questionnaires and there was no significant different difference between those under and over 60 years of age.

**Conclusions:**

Our findings suggest that the ECQ is not as sensitive or specific a diagnostic tool in 1^st ^generation Black African-Caribbean and South Asian UK migrants than in the Edinburgh Artery Study, reflecting the findings of other diagnostic questionnaires in these minority ethnic groups. However this study is limited by sample size so conclusions should be interpreted with caution.

## Background

Peripheral artery disease (PAD) is an important healthcare problem in developed nations and is associated with considerable morbidity and mortality. Intermittent claudication (IC) is the most common symptomatic manifestation of PAD, and typically occurs in up to one third of patients with this disease [1]. Intermittent claudication is characterised by pain, aching or cramping in the calf, buttock, hip or thigh on ambulation that resolves upon rest. Symptoms arise from an inadequate blood supply to the peripheral arteries of the legs that result in anaerobic metabolism and build up of lactic acid within the muscles. Only about a quarter of patients with IC will ever significantly deteriorate [1].

Ankle brachial pressure index (ABPI) is the gold standard for the assessment of both asymptomatic and symptomatic PAD [2]. A value of <0.9 is indicative of PAD, with sensitivity and specificity of 95% for detecting angiogram positive disease [3-5]. Intermittent claudication can be diagnosed with the use of a questionnaire along with evidence of PAD. The Edinburgh Claudication Questionnaire (ECQ) was first validated by Leng et al. 1992 after noting that the previous WHO/Rose questionnaire had low sensitivity [6] This patient administered questionnaire was administered to a predominantly European population and was found to be 91.3% sensitive and 99.3% specific for IC in comparison to a doctor made diagnosis [6]. It was also found to have excellent reliability after repeating the questionnaire at 6 months. The ECQ has been validated in French and Brazilian Portugese [7-9] and in English in a community based study in the Netherlands [10] though not amongst languages of the Indian Sub-continent or amongst Black African-Caribbean groups.

Ethnic minority groups make up 7.9% of the general population of the UK [11]. The largest of these being Asian/Asian British (50.2%) and Black/Black British (24.8%) [11]. Studies have shown that questionnaires designed to diagnose cardiovascular diseases in European populations may not always be applicable in an ethnically diverse population [12,13]. In order to meet the healthcare needs of the diverse populations which exists in the UK [11], it is important to know if any differences exist in the reporting of symptoms of disease and also whether current diagnostic tools designed in European populations are applicable in the diagnosis of disease in other ethnic groups.

The purpose of this sub-study to the Ethnic-Echocardiographic Heart of England Screening Survey (E-ECHOES) [14] was to determine the diagnostic accuracy of the ECQ to diagnose PAD, defined by ABPI <0.9, in languages of the Indian sub-continent and also in English speaking Black African-Caribbean groups, with a view to their use or adaptation in a follow up epidemiological survey.

## Methods

### Translation of Edinburgh Claudication of Questionnaire

**Figure 1:**
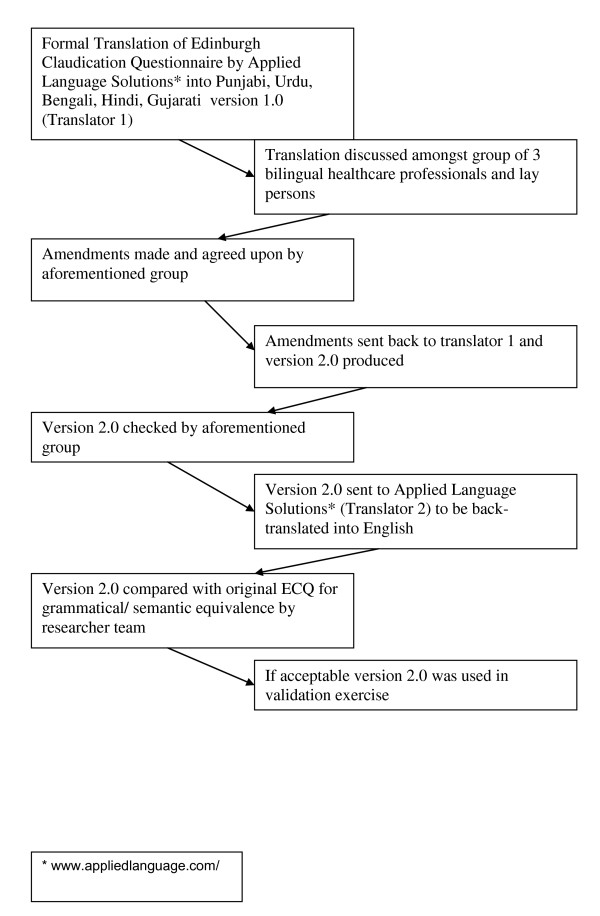
**Translation process of the Edinburgh Claudication Questionnaire**.

A diagrammatic representation of the translation process for each South Asian language is shown in Figure 1. The original ECQ was formally translated into each of Hindi, Punjabi, Gujarati, Urdu and Bengali (Additional file 1). For each translation a consortium comprising 3 bilingual healthcare professionals and a lay person was used to assess grammatical and semantic equivalence. A general consensus was made between these 4 people as to whether amendments needed to be made to the original translation. If so, the suggested amendments were sent back to the initial independent translator and a new version was produced. Once a translation was deemed to be acceptable, it was then independently back-translated into English. The back-translated version was then compared to the original English version of the ECQ. All translations were found to be grammatically and semantically equivalent. Translated versions of the ECQ are shown in appendix 1.

### Study Design &Recruitment

This was a sub-study of the E-ECHOES UK community survey screening South Asian and Black African-Caribbean residents of Birmingham aged 45 years or over for heart failure. The design of the E-ECHOES study has been reported previously, and we provide a brief summary [14]. Using 2001 Census data, wards serving the Birmingham Special Health Authority area, having >50% Black and minority Ethnic Groups as residents were selected and 20 practices recruited from these wards. Using the practice age-sex register, all subjects of South Asian or Black ethnicity age 45 and over were invited to participate. Subjects attended for an assessment at their local general practice. All participants screened between October 2007 and February 2009 were asked to complete the ECQ in their chosen language. Participants answering 'yes' to question one were subsequently invited back for ABPI assessment. The study was approved by the local research and ethics committee and written informed consent was obtained from all patients.

### Questionnaire Validation

Patients attending for assessment as part of E-ECHOES were asked to complete the ECQ in their preferred language, including English. All literate patients completed the questionnaire independently and illiterate patients were provided with a bilingual interpreter if required and the questions were read out to them as written. Participants responding negatively to question one of the ECQ ("Do you get a pain or discomfort in your leg(s) when you walk?") in any language were not included in the analysis.

### PAD Assessment

The presence of PAD was assessed through the measurement of ABPI which was measured at rest in the supine position with a hand held 8 MHz Doppler (Huntleigh Super Dopplex II) and a manual sphygmomanometer. Systolic blood pressure (SBP) in the brachial artery was measured in both arms using an appropriately sized blood pressure cuff and Doppler detection in the antecubital fossa. SBP was recorded 3 times in each arm, and in the left and right dorsalis pedis and posterior tibial arteries just proximal to the malleoli. For each pressure measurement, the pulse was located using the Doppler probe and the cuff then inflated until the pulse was obliterated. The cuff was then deflated slowly and the pressure noted when the pulse detected by the Doppler probe re-appeared. ABPI was calculated for each leg as the ratio of the higher of the two SBPs at the ankle and the average of the left and right brachial SBP, unless there was a discrepancy ≥10 mmHg in blood pressure values between the 2 arms, in which case the higher side SBP was used. To standardise the blood pressure measurements all recordings were performed by 1 operator (PB), trained in the measurement of ABPI.

ABPI values ≤0.9 in one or both legs were considered diagnostic of PAD. The absence of PAD was defined as levels from 0.91 - 1.4 in the absence of re-vascularisation of the lower limbs. ABPI values >1.4 were excluded from the analysis as they do not define the diagnosis of PAD.

### Results of the Edinburgh Claudication Questionnaire

The diagnosis of a positive questionnaire was made on the basis of the original guidelines - see additional file 1. The respondent must have answered *yes *to question 1, *no *to question 2, *yes *to question 3, *usually disappears in less than 10 minutes *to question 5 and in question 6, mark the calf, thigh or buttock regions. A negative questionnaire was one that did not have this exact combination. Question 4 was only used to define the severity of claudication if present.

### Definition of Intermittent Claudication

A positive questionnaire along with an ABPI <0.9 would be diagnostic of intermittent claudication for the purpose of this study.

### Statistical analysis

Questionnaire performance was assessed using Minitab 15 (State Coll, PA). The sensitivity, specificity, positive predictive value, negative predictive values were calculated. Diagnostic accuracy was then calculated by dividing the number of individuals under correct classification on the ECQ by the total number of subjects assessed.). Data with a continuous variation were subjected to the Anderson-Darling test to determine mode of distribution. If normally distributed, such data is summarised using mean and standard deviation, and if non-normally distributed by median inter-quartile range. One way ANOVA was used to assess whether there were any differences in continuous variables between the 3 groups (speakers of English, Punjabi or Bangladeshi). Fisher's exact test was used in 2 × 2 tables between participants with ABPI <0.9 and participants without for each question of the ECQ to determine whether any particular question was responsible for contributing to the differences in overall sensitivity and specificity of the study questionnaires. Significance was defined as p < 0.05.

## Results

All Participants (n = 2831) screened as part of E-ECHOES between October 2007 and February 2009 were asked to complete the ECQ in their chosen language. 154 participants (5.4%) answering positively to question one "Do you get pain or discomfort in your leg(s) when you walk? were invited to attend for a subsequent assessment of ABPI of which, 74.3% of eligible participants attended. Demographic data for attenders and non-attenders is shown in Table 1. Non-attendees were significantly younger (59 standard deviation [SD][9] vs. 65 [11] years; p = 0.015) and had significantly more Gujarati speakers (14.3 vs. 1.8%; p = 0.003). There were no differences in cardiovascular risk factors and illiteracy rate between those participating in validation exercise and those not.

**Table 1:** Characteristics of Participants Eligible for participation in Validation Study

Variable	ABPI attendees (n = 113) [SD]	Non-attendees (n = 41) [SD]	p-value
Age	65 [11]	59 [9]	0.015
Male (%)	53	52	0.916
Illiteracy (%)	29.5	31	0.875
**Language**			
Bengali (%)	17.7	10.7	0.371
English (%)	32.7	46	0.175
Guajarati (%)	1.8	14.3	0.003
Punjabi (%)	32.7	25	0.429
Urdu (%)	15	3.6	0.088
mean SBP (mmHg)	138 [25]	135 [16]	0.62
mean DBP (mmHg)	77 [12]	79 [8]	0.585
BMI	29 [5]	31 [5]	0.06
Ever Smoker (%)	56.6	58.6	0.846
Current Smoker (%)	15.3	31	0.057
Hypertensive (%)	65.5	69	0.732
Diabetic (%)	50	34.5	0.145
CAD (%)	35.5	25	0.301
CBVD (%)	16.1	13.8	0.762

SD: Standard deviation; SBP: systolic blood pressure; DBP: diastolic blood pressure; BMI: body mass index; CAD: coronary artery disease; CBVD: cerebrovascular disease

**Figure 2:**
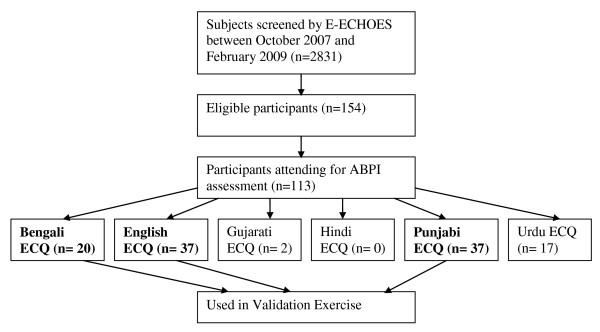
**Recruitment of subjects**.

The total number of respondents completing each version of the ECQ is illustrated in Figure 2. All participants completing the ECQ in English were 1^st ^generation Black Caribbeans. All participants completing the translated versions were 1^st ^generation South Asian migrants. Of the 6 languages, only English, Punjabi and Bengali had participants with ABPI <0.9 and ECQ suggestive of claudication, so these were used in the questionnaire validation in this pilot study.

The mean age of participants in this study was 65 years of whom 53% were males and 29.5% were illiterate. PAD was evident in 21.2% [95% CI: 14.7-29.7] of subjects with a positive response to ECQ question one and subsequently invited back for ABPI assessment. Intermittent claudication was present in 8.8% [95% CI: 4.9-15.5] of these participants. There was no significant age difference between those with PAD and those without. The participant demographics of those completing each version of the ECQ are shown in Table 2.

**Table 2:** Participant Demographics by language of Edinburgh Claudication Questionnaire

Variable	English* (±SD) [IQR] n = 37	Punjabi (±SD) [IQR] n = 37	Bangladeshi (±SD) [IQR] n = 20	p-value
Age (years)	67 (9)	66 (13)	64 (9)	0.577
% Male	55.9	41.7	66.7	0.192
% Illiteracy	0	45.9	50	<0.0001
Age of leaving school	16 [15-17]	15 [9-17]	11 [8-15]>	0.648
% Higher education	2.7	2.7	5	0.874
% ABPI <0.9	32.4	16.6	20	0.237
% IC	16.2	8.1	5.0	0.345
ABPI	0.95	1.03	0.98	0.155
Diabetes mellitus (%)	65.4	34.5	58.8	0.062
Hypertension (%)	73.9	60	70.6	0.532
Ever Smoker (%)	61.1	40.5	57.9	0.182
Current Smoker (%)	14.8	20.6	10.5	0.616
Coronary artery disease (%)	37	28.1	52.6	0.216
Cerebrovascular disease (%)	24.1	9.4	11.8	0.251
Body Mass Index	30.4 (5.5)	28.2 (5.4)	28.2 (4.3)	0.271
Waist circumference (cm)	92.6 (14.8)	94 (13.9)	97.4 (11.5)	0.488

SD: standard deviation; ABPI: ankle brachial pressure index; IC: intermittent claudication; * 1^st ^Generation Black Caribbean UK migrants. Data presented as mean (SD) or percentage.

### Punjabi Questionnaire

Thirty-seven participants completed this translated version (Table 2). Their mean age was 66 [s.d. 13] years and 41.7% were male. 49.5% were illiterate and required a bilingual translator to read and interpret the participants' responses. For subjects attending school, the median age of leaving education was 15 [inter-quartile range (IQR) 9-17] years, with only 2.7% attending higher education. The sensitivity of the Punjabi ECQ was 50%, specificity 87%, positive predictive value (PPV) 43% and negative predictive value 90% (Table 3). The diagnostic accuracy of this version was 83.8%. We attempted to look for differences in sensitivity and specificity between illiterate (sensitivity 33.3%; specificity 92.9%) and literate (sensitivity 66.6%; specificity 86.7%) participants. There were no differences in diagnostic accuracy between these 2 groups (84.2 vs.83.3%). We also attempted to investigate whether age would affect the sensitivity and specificity of the ECQ and found participants under the age of 60 years had higher sensitivity (100 vs. 50%) but lower specificity (83.3 vs.93%) than those older than 60 years. However due to very small numbers of true claudicants significance was not reached. No differences in diagnostic accuracy between those under or over 60 years were reported (<60 years: 84.6%; >60 years: 84.2%).

**Table 3:** Sensitivity, Specificity, Positive and Negative predictive values of the Edinburgh Claudication Questionnaire

Language	Sensitivity (%) [95% CI]	Specificity (%) [95% CI]	PPV (%) [95% CI]	NPV (%) [95% CI]
English*	50 [22-78]	68 [50-86]	43 [16-68]	74 [55-91]
Punjabi	50 [10-90]	87 [75-99]	43 [5-79]	90 [79-100]
Bangladeshi	33.3 [0-86]	50 [26-75]	13 [0-31]	73 [45-99]

* 1^st ^Generation Black Caribbean UK migrants; PPV: positive predictive value; NPV: negative predictive value; CI: confidence interval

### Bengali Questionnaire

Twenty participants completed the Bengali translation (Table 2), in whom the mean age was 64 [s.d. 9] years and 66.7% were male. 50% were illiterate, and required a bilingual translator to complete the questionnaire, and of those attending school the median age of leaving was 11 [IQR [8-15]] years. Only 5% attended higher education. The sensitivity and specificity of this translation were 33.3% and 50% respectively and PPV and NPV were 13% and 73% respectively (Table 3). The diagnostic accuracy of the Bengali ECQ was 45%. None of the illiterate Bengali speakers had intermittent claudication and so we could not compare the sensitivity of this questionnaire between those attending school and those whom never attended. The specificity between the former and latter were 66.7 and 50% respectively bit this failed to reach statistical significance. Likewise no Bengali participants below the age of 60 had intermittent claudication and no significant difference in sensitivity was found with age. Diagnostic accuracy did not differ significantly between the 2 groups (<60 years: 50%; >60 years: 58.3%)

### English Questionnaire

Thirty-seven participants completed the original English version of the ECQ (Table 2), all of whom were African Caribbean. Their mean age was 67 [s.d. 9] years and 55.9% were male. All participants attended school and the median age of leaving school was 16 [IQR 15-17] years. 2.7% attended higher education. Sensitivity and specificity were 50% and 68% respectively and PPV and NPV were 43% and 74% (Table 3). The diagnostic accuracy of this version was 62.2%. The sensitivity of the ECQ in those under 60 and over 60 years of age were 50 vs. 55.6% respectively and specificity were 100 vs. 75% respectively but results were not significant due to the low number of participants. The diagnostic accuracy was 66.7% for <60 years and 56.7% >60 years of age.

### Overall Cohort

There was no difference in age, sex distribution and prevalence of cardiovascular risk factors between the English, Punjabi and Bengali ECQ groups (Table 1). There were also no differences in body mass index and waist circumference in these 3 groups. There were significant differences in illiteracy between South Asian participants and African Caribbean participants (Table 2). Of those attending school however, no significant differences were found in median age of leaving and proportion of people attending higher education.

We investigated the sensitivity and specificity of each of the questions 2 to 6 in the ECQ (Table 4). In all languages question 3, "Do you get it [Pain] when you walk uphill or hurry?" was the most sensitive. The least sensitive question was question 5, pertaining to duration of pain. This question was overall the most specific in the diagnosis of intermittent claudication.

**Table 4:** Sensitivity, Specificity, Positive and Negative predictive values of each question of the Edinburgh Claudication Questionnaire

ECQ Question	Sensitivity (%)	Specificity (%)	PPV (%)	NPV (%)
**English (n = 37)**				
**Q2**	25	44	8	75
**Q3**	100	20	19	100
**Q4**	75	44	20	90
**Q5**	41.7	48	13	81
**Q6**	66.7	20	14	76
**Punjabi (n = 37)**				
**Q2**	50	45.2	7	91
**Q3**	100	32.3	8	1
**Q4**	100	32.3	11	1
**Q5**	66.7	64.5	14	96
**Q6**	83.3	22.6	9	94
**Bengali (n = 20)**				
**Q2**	75	62.5	10	98
**Q3**	100	62.5	5	100
**Q4**	75	40	6	97
**Q5**	25	50	3	93
**Q6**	100	62.5	5	1

## Discussion

We have shown that translated versions of the ECQ into South Asian languages and the original English version in 1^st ^generation Black Caribbean migrants have lower sensitivity and specificity than the original version [6] but similar levels reported in other populations [7,10]. We also report significant differences in diagnostic accuracy between the Punjabi, Bengali and English versions. Our study differs from the study by Leng et al. in that we used ABPI, an objective measure, rather than a doctor made diagnosis of PAD.

The ECQ was developed and validated as part of the Edinburgh Artery Study [6] with the objective of improving the sensitivity of the WHO/Rose Claudication Questionnaire [15]. The researchers questioned 300 participants over the age of 55 with leg pain and reported 91.3% sensitivity and 99.3% specificity of the ECQ at diagnosing intermittent claudication. The original ECQ was used in large observational study investigating people presenting to their general practitioner with symptoms suggestive of IC, in the Netherlands, which reported a much lower sensitivity of 56.2% [10]. Makdisse et al. recently published a Brazilian Portuguese version of the ECQ and reported 85% sensitivity and 93% specificity [9]. Previously Aboyans et al. published a French version with 86.5% sensitivity and 95.6% specificity [8] which was subsequently repeated by Lacroix et al., who reported a marked difference in the questionnaire's sensitivity at 47% [7].

Studies have previously shown that questionnaires designed to diagnose cardiovascular disease in White European populations may not always be applicable in an ethnically diverse population [12,13,16]. Our preliminary findings suggest the ECQ is not a sensitive or specific diagnostic tool for IC in Black African-Caribbean and South Asian groups. This reflects the findings of other researchers using the Rose Angina questionnaire [12,13]. It is possible that South Asians and Blacks are less good at describing pain than white European populations, which may account for the apparent differences in sensitivity and specificity when compared to Leng et al. [6]. Indeed it has previously been reported that the Rose Angina questionnaire has a lower sensitivity and specificity in South Asians than in white Europeans [12]; site of pain and duration of pain being least likely to score a positive response to Rose Angina questionnaire in both South Asian men and women. We found question 5 of the ECQ, pertaining to duration of pain to have the least specificity of all of the questions in the ECQ in all versions, which may partly explain the low sensitivity and specificity we found overall. People of African descent have also been reported to be less likely to score positively to angina using the Rose questionnaire and less likely to seek treatment than white group [16].

We used an objective measure (ABPI) to diagnose PAD rather than clinical assessment only, and our findings of lower sensitivity and specificity, positive and negative predictive values of the ECQ when compared to Leng et al. are comparable to other population surveys [7,10]. The Edinburgh artery study used clinical assessment, rather than ABPI, to diagnose PAD and as such there would be an increased chance of matching ECQ results with the presence of PAD as both diagnoses would have focussed on symptomatic disease. Using an objective measure such as ABPI would put patients with ABPI < 0.9 but ECQ negative as being false positives and patients representing symptoms of IC (ECQ positive) but ABPI > 0.9 would be classed as false negatives, which would lower the sensitivity and specificity and overall accuracy of the questionnaire in its current form.

### Limitations

The main limitations to this study were recruitment of participants answering positively to question one of the ECQ and re-attendance for ABPI measurement. Previous studies used more symptomatic participants in their validation exercises [9,10]. However, as the E-ECHOES study [14] was based within primary care and screened all eligible subjects whether or not they were symptomatic with cardiovascular disease; only 5.4% of the 2831 E-ECHOES participants were eligible to take part in this sub-study. The attempt to validate the ECQ in several languages meant the number of expected cases of IC in each language questionnaire was going to be low. Of the eligible participants, only 74.3% returned for ABPI assessment. This may have resulted in responder bias and may have affected the validity of the ECQ. We analysed potential differences in participant characteristics between non-attendees and those participating in the validation exercise and suggest that the significantly younger age of the former group may have resulted in work-related commitments preventing a return visit for ABPI measurement. However, the younger age group might also have had a lower risk of PAD and therefore may not have yielded more cases of IC. It is possible the inclusion of illiterate participants may have contributed to the low sensitivity, specificity and diagnostic accuracy of the ECQ versions. However, we found no significant differences in these groups. It is also possible that the age of participants may have contributed to the diagnostic accuracy of the questionnaires. However we found no significant differences. Our findings must be interpreted with caution however due to aforementioned sample size limitations.

Another limitation is that we used ABPI alone as a diagnostic tool for PAD, rather than confirming the diagnosis with imaging. As such, subjects with arterial calcification may have had falsely elevated ABPI, contributing to some of the false negative results, which may have masked an underlying diagnosis of claudication. Other diseases potentially affecting our population, such as Takayasu disease, would not have been detected by ABPI alone and as such may have also contributed to the false negatives we found.

## Conclusions

This validation study findings suggest the original English ECQ is not a sensitive or specific tool in the diagnosis of intermittent claudication in UK Black African Caribbean migrants. Punjabi and Bengali versions also did not show high sensitivity and specificity. Larger studies, involving minority ethnic groups, need to be performed before firm conclusions can be made about the utility of the ECQ in non-White groups. The high concordance between cardiovascular risk factors and leg pain reported in this sub-study should prompt clinicians to perform an objective assessment of PAD, such as ABPI, in suspicious patients presenting to primary and secondary care. Subjects with ABPI <0.9 should undergo further investigations to confirm the diagnosis and if appropriate, should receive optimal medical management.

## Competing interests

The authors declare that they have no competing interests.

## Authors' contributions

PCB, GHYL and PSG conceived the study that was developed with SS and ADB. PCB undertook measurements, statistical analysis and wrote the first draft. All authors read, commented and approved the final version. PSG is guarantor.

## Pre-publication history

The pre-publication history for this paper can be accessed here:

http://www.biomedcentral.com/1471-2288/11/85/prepub

## Supplementary Material

Additional file 1 **The Edinburgh Claudication Questionnaire (ECQ) **[6]. This file contains the translated versions of the Edinburgh Claudication Questionnaire into Bengali, Gujarati, Hindi, Punjabi, and Urdu. Click here for file
