# Supplementary material for: Validation of the Edinburgh Claudication Questionnaire in 1st generation Black African-Caribbean and South Asian UK migrants: A sub-study to the Ethnic-Echocardiographic Heart of England Screening (E-ECHOES) study
Source: BMC Med Res Methodol. 2011 Jun 3;11:85. doi: 10.1186/1471-2288-11-85 (PMC3128858; doi:10.1186/1471-2288-11-85)

# Additional file

**The Edinburgh Claudication Questionnaire (ECQ) [6]**

(a) ECQ – English version

(1) Do you get a pain or discomfort in your leg(s) when you walk? Yes ⁭ No ⁭

If you answered "Yes" to question (1), please answer the following questions. Otherwise you need not continue

(2) Does this pain ever begin when you are standing still or sitting? Yes ⁭ No ⁭

(3) Do you get it if you walk uphill or hurry? Yes ⁭ No ⁭

(4) Do you get it if you walk at an ordinary pace on the level? Yes ⁭ No ⁭

(5) What happens to it if you stand still?

- 1. usually continues for more than 10 minutes ⁭
  2. usually disappears in 10 minutes or less ⁭

(6) Where do you get this pain or discomfort?

Mark the place (s) with an ‘X’ on the diagrams below

Front Back


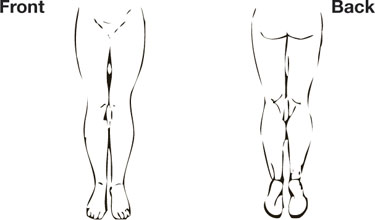


Definition of positive classification requires all of the following responses: "Yes" to (1), "No" to (2), "Yes" to (3), grade 1 "No" to (4), grade 2 "Yes" to (4). If these criteria are fulfilled, a definite claudicant is one who indicates the pain is in the calf, regardless of whether pain is also marked at other sites; a diagnosis of atypical claudication is made if the pain is marked in the thigh or buttock, in the absence of any calf pain. Subjects should not be considered to have claudication if pain is indicated in the hamstrings, feet, shins, joints or appears to radiate in the absence of any pain in the calf

(b) ECQ- Bengali Translation


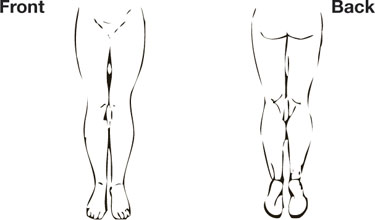


(c) ECQ- Gujarati Translation


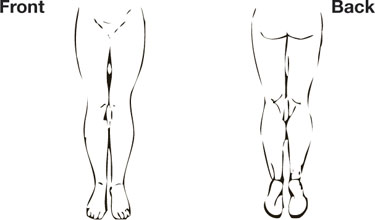


(d) ECQ- Hindi Translation


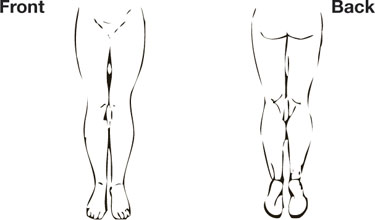


(e) ECQ- Punjabi Translation


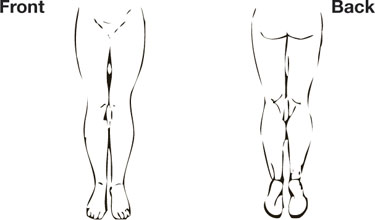


(f) ECQ- Urdu Translation


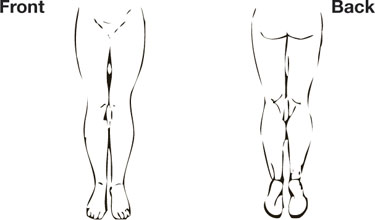

Supplement: Additional file 1 — The Edinburgh Claudication Questionnaire (ECQ) [6]. This file contains the translated versions of the Edinburgh Claudication Questionnaire into Bengali, Gujarati, Hindi, Punjabi, and Urdu. [file 1471-2288-11-85-S1.DOC]
